# Treatment strategy and prognostic factors for Krukenberg tumors of gastric origin: report of a 10-year single-center experience from China

**DOI:** 10.18632/oncotarget.19759

**Published:** 2017-08-01

**Authors:** Pengfei Yu, Ling Huang, Guoping Cheng, Litao Yang, Gaiguo Dai, Jieer Ying, Yian Du

**Affiliations:** ^1^ Department of Abdominal Surgery, Zhejiang Cancer Hospital, Hangzhou 310022, China; ^2^ Department of Pathology, Zhejiang Cancer Hospital, Hangzhou 310022, China; ^3^ Department of Medical Oncology, Zhejiang Cancer Hospital, Hangzhou 310022, China

**Keywords:** Krukenberg tumors, gastric cancer, metastasectomy, chemotherapy, prognosis

## Abstract

**Background:**

Gastric cancer patient with ovarian metastasis is common in clinical practice, but it is still uncertain whether surgical resection of ovarian metastasis could improve the outcome. This study aimed to explore the survival benefit of metastasectomy plus chemotherapy over chemotherapy alone in the treatment of Krukenberg tumors arising from gastric cancer and to identify prognostic factors.

**Results:**

A total of 152 patients were identified, including 93 patients with synchronous ovarian metastasis and 59 patients with metachronous ovarian metastasis. Overall survival (OS) was significantly better in metastasectomy group relative to the non-metastasectomy group for patients with synchronous ovarian metastasis (19.0 months vs. 11.8 months; *P* < 0.001) and those with metachronous ovarian metastasis (24.6 months vs. 14.3 months; *P* = 0.02), respectively. Metastasectomy (hazard ration [HR] 0.486; 95% confidence interval [CI] 0.323–0.729; *P* < 0.001), peritoneal carcinomatosis (HR 1.934; 95% CI 1.230–3.049; *P* = 0.004), and expression status of ER-β (HR 0.404; 95% CI 0.251–0.648; *P* < 0.001) and PR (HR 0.496; 95% CI 0.301–0.817; *P* < 0.001) were independent predictors of OS.

**Methods:**

All patients who were diagnosed with gastric cancer and ovarian metastases between January 2005 and December 2014 were included in the current study. Patients were subdivided according to treatment modality: the metastasectomy group (metastasectomy plus chemotherapy) and the non-metastasectomy group (chemotherapy alone). The clinicopathological features and the treatment records were reviewed in detail and their association with survival were analyzed.

**Conclusion:**

Metastasectomy plus chemotherapy was associated with survival benefits in patients with Krukenberg tumors from gastric cancer. Metastasectomy, peritoneal carcinomatosis, and expression status of ER-β and PR were independent prognostic factors for survival.

## INTRODUCTION

Gastric cancer is one of the most common cancers worldwide, and the incidence is particularly high in Asian countries, including China [1]. Metastasis and recurrence are the major causes for poor prognosis in gastric cancer. Ovarian metastasis (Krukenberg tumor) is usually seen in female patients, including synchronous metastasis, and metachronous metastasis after curative resection of gastric cancer [2]. The reported incidence of ovarian metastasis or Krukenberg tumor is approximately 0.3% to 6.7%, however, some autopsy studies have reported incidence rates of 33% to 41% [3, 4]. Ovarian metastasis is associated with poor prognosis, and is one of the most important causes of treatment failure for gastric cancer in female patients [5]. Although systemic chemotherapy can provide symptom palliation and prolonged survival in patients with ovarian metastasis, the efficiency and survival time remain disappointing [2, 6]. Several studies have explored the utility of metastasectomy for Krukenberg tumors in patients with gastric cancer [7, 8] However, the role of ovarian metastasectomy is still under debate and is likely to benefit only a specific subset of patients [9]. So far, the optimal treatment strategy for Krukenberg tumors from gastric cancer had not been clearly established. This study was conducted to determine associations between metastasectomy of Krukenberg tumors, clinicopathological features, and survival outcome, and consequently provide optimal treatment strategy for these patients.

## RESULTS

### Patient characteristics

The median follow-up duration for all patients was 37.2 (range 2.5–71) months; median age at treatment onset was 43.4 (range 18–65) years, and mean size of metastatic ovarian tumors was 8.13 (range 2–20) cm.

Clinicopathologic characteristics of gastric cancer with synchronous or metachronous ovarian metastasis are listed in Tables 1 and 2, respectively. Of these, tumor location, differentiation, ascites, ER and PR expression, tumor markers, and TNM stage were similar for both groups of either synchronous or metachronous ovarian metastasis.

**Table 1:** Clinical characteristics of 93 patients with synchronous Krukenberg tumors

Variable	Metastasectomy plus chemotherapy (*n =* 49)	Chemotherapy alone (*n =* 44)	*P*-value
Median age	43.1 (26–65)	40.9 (18–61)	0.290
Tumor size (cm)	8.70 (2–20)	7.33 (2.2–18)	0.119
ECOG performance status			
0-1	47	40	0.326
2	2	4	
Laterality			
Bilateral	33	31	0.747
UnilateraI	16	13	
Peritoneal metastasis			
No	35	37	0.145
Yes	14	7	
Signet-ring cells			
Positive	36	29	0.427
Negative	13	15	
Differentiation			
Well and moderately	8	4	0.299
Poorly	41	40	
Ascites			
No	20	11	0.106
Yes	29	33	
ER			
Positive	22	14	0.196
Negative	27	30	
PR			
Positive	16	8	0.111
Negative	33	36	
Serum CEA (ng/mL)			
Normal	40	36	0.982
>5	9	8	
Serum CA19-9 (U/mL)			
Normal	33	26	0.409
>39	16	18	
Serum CA125 (U/mL)			
Normal	20	14	0.368
>35	29	30	

**Table 2:** Clinical characteristics of 59 patients with metachronous Krukenberg tumors

Variable	Metastasectomy plus chemotherapy (*n =* 40)	Chemotherapy alone (*n =* 19)	*P*-value
Median age	46.9 (31–62)	42.7 (20–60)	0.128
Tumor size (cm)	8.78 (4.3–20)	7.73 (4.5–16.7)	0.261
ECOG performance status			
0-1	39	17	0.190
2	1	2	
Laterality			
Bilateral	32	16	0.700
UnilateraI	8	3	
Peritoneal metastasis			
No	29	15	0.595
Yes	11	4	
Signet-ring cells			
Positive	31	16	0.550
Negative	9	3	
Differentiation			
Well and moderately	6	4	0.563
Poorly	34	15	
AJCC stage			
I–II	5	1	0.390
III–IV	35	18	
Ascites			
No	9	5	0.748
Yes	31	14	
ER			
Positive	17	8	0.977
Negative	23	11	
PR			
Positive	13	6	
Negative	27	13	0.944
Serum CEA (ng/mL)			
Normal	33	16	0.870
>5	7	3	
Serum CA19-9 (U/mL)			
Normal	31	13	0.454
>39	9	6	
Serum CA125 (U/mL)			
Normal	21	7	0.260
>35	19	12	

### Treatment outcome and prognostic factors

**Figure 1:**
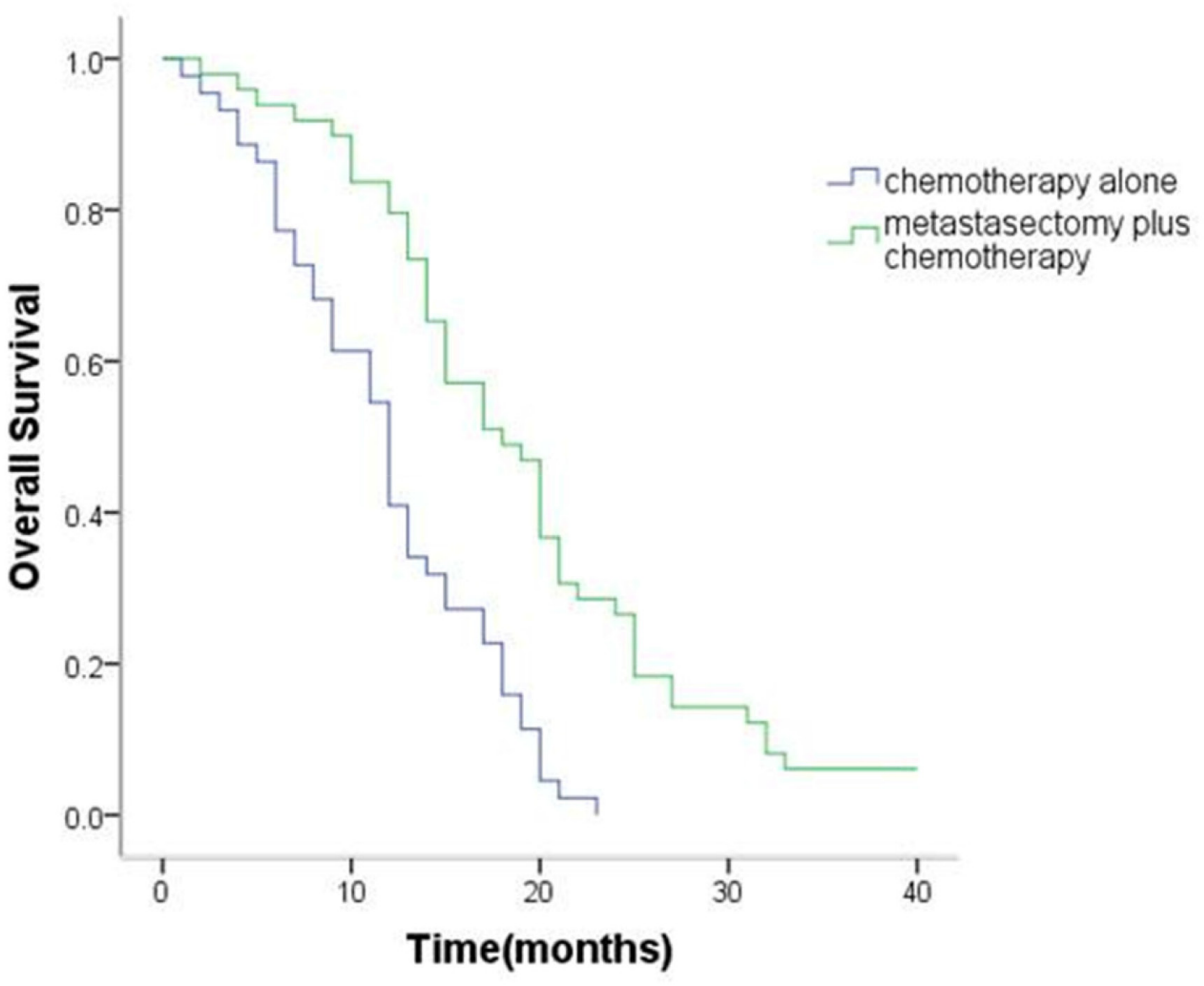
Kaplan–Meier analysis of overall survival in patients with or without metastasectomy of synchronous Krukenberg tumors

**Figure 2:**
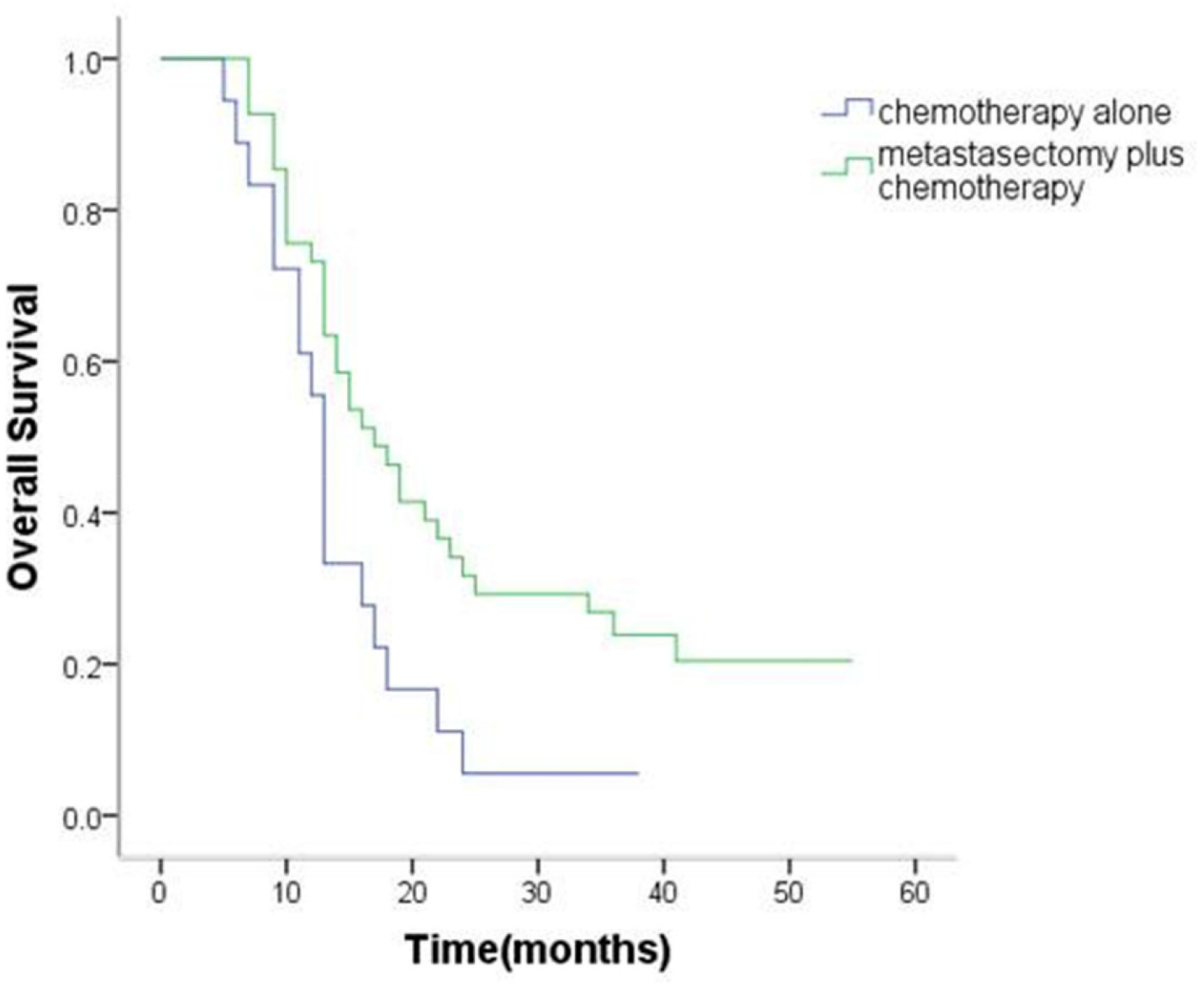
Kaplan–Meier analysis of overall survival in patients with or without metastasectomy of metachronous Krukenberg tumors

The median OS of patients with synchronous ovarian metastasis was 15.6 months (95% confidence interval [CI], 13.9 to 17.3 months). The median OS of metastasectomy group and non-metastasectomy group was 19.0 months (95% CI,16.6 to 21.5 months) and 11.8 months (95% CI, 10.1 to 13.5months), respectively. For patients with metachronous ovarian metastasis, median OS was 21.7 (95% CI 17.6–25.9) months. The median OS of metastasectomy group and non-metastasectomy group was 24.6months (95% CI,19.3 to 30.0 months) and 14.3 months (95% CI, 10.8 to 17.8months), respectively. Therefore, patients in the metastasectomy group had a significantly better OS than patients in the non-metastasectomy group (synchronous ovarian metastasis: P < 0.001, Figure 1; metachronous ovarian metastasis: P = 0.02, Figure 2).

Based on univariate analysis, metastasectomy, peritoneal carcinomatosis, signet ring cell, ascites, expression of ER-β and PR, and serum levels of CA19-9 (>37 U/mL) were prognostic factors associated with survival. After adjusting covariates on multivariate analysis, metastasectomy (hazard ration [HR] 0.486; 95% CI 0.323–0.729; *P* < 0.001), presence of peritoneal carcinomatosis (HR 1.934; 95% CI 1.230–3.049; *P* = 0.004), ER-β positivity (HR 0.404; 95% CI 0.251–0.648; *P* < 0.001), and PR positivity (HR 0.496; 95% CI 0.301–0.817; *P* < 0.001) were independent predictors of OS (Table 3).

**Table 3:** Univariate and multivariate analysis of overall survival

Variable	Univariate		Multivariate	
	HR (95% CI)	*P*-value	HR (95% CI)	*P*-value
Age (≥50 years)	0.712 (0.445–1.139)	0.156	-	-
Size of tumor (<5 cm)	0.736 (0.491–1.104)	0.139	-	-
Bilateral ovarian metastases	0.775 (0.515–1.165)	0.220	-	-
Metastasectomy	0.467 (0.318–0.685)	<0.001	0.486 (0.323–0.729)	<0.001
Peritoneal carcinomatosis	2.359 (1.585–3.512)	<0.001	1.934 (1.230–3.049)	0.004
Signet-ring cells	1.871 (1.272–2.751)	0.001	1.183 (0.778–1.802)	0.430
Ascites	1.968 (1.269–3.051)	0.002	1.450 (0.914–2.294)	0.114
Gastrectomy	0.761 (0.520–1.114)	0.160	-	-
ER positive	0.254 (0.166–0.389)	<0.001	0.404 (0.251–0.648)	<0.001
PR positive	0.376 (0.238–0.596)	<0.001	0.496 (0.301–0.817)	<0.001
CA125	1.427 (0.939–2.170)	0.096	-	-
CEA	1.369 (0.873–2.146)	0.171	-	-
CA199	1.628 (1.108–2.393)	0.013	1.447 (0.963–2.179)	0.075

**Figure 3:**
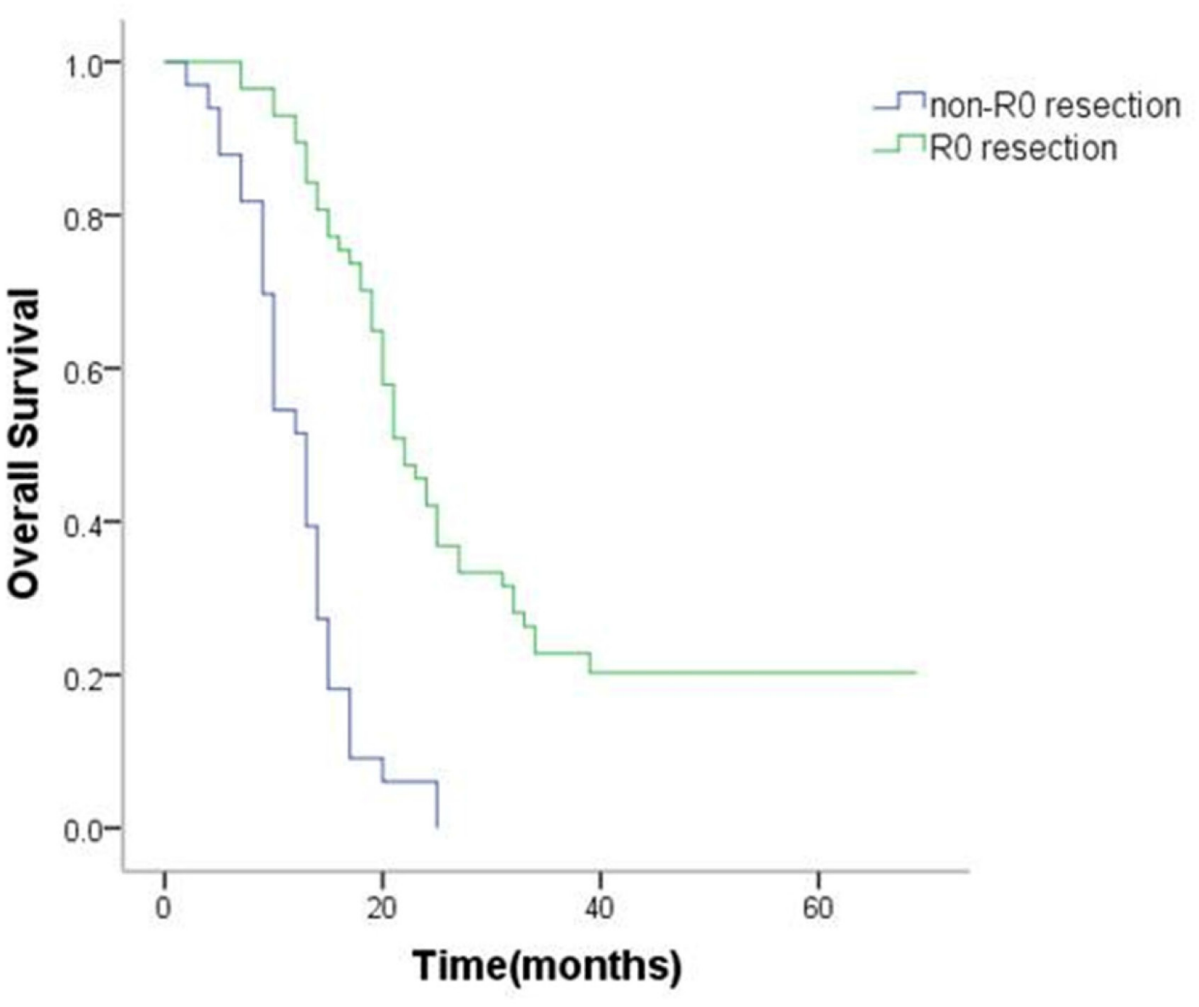
Kaplan–Meier analysis of overall survival in patients with or without R0 resection

Additionally, 63.3% (57/90) of the patients had R0 resection. Median OS was 30.5 (95% CI 25.1–35.9) months in the R0 resection group and 12.2 (95% CI 10.4–14.0) months in the non-R0 resection groups. Survival was superior in the R0 resection group as compared to the non-R0 resection groups (*P* < 0.001; Figure 3).

### Correlation between expression of sex hormone receptors, clinicopathologic factors, and survival

The positive rate of ER-β and PR was 44.7% and 31.6%.However, ER-α showed no positivity in any gastric cancer tissue sample.

**Figure 4:**
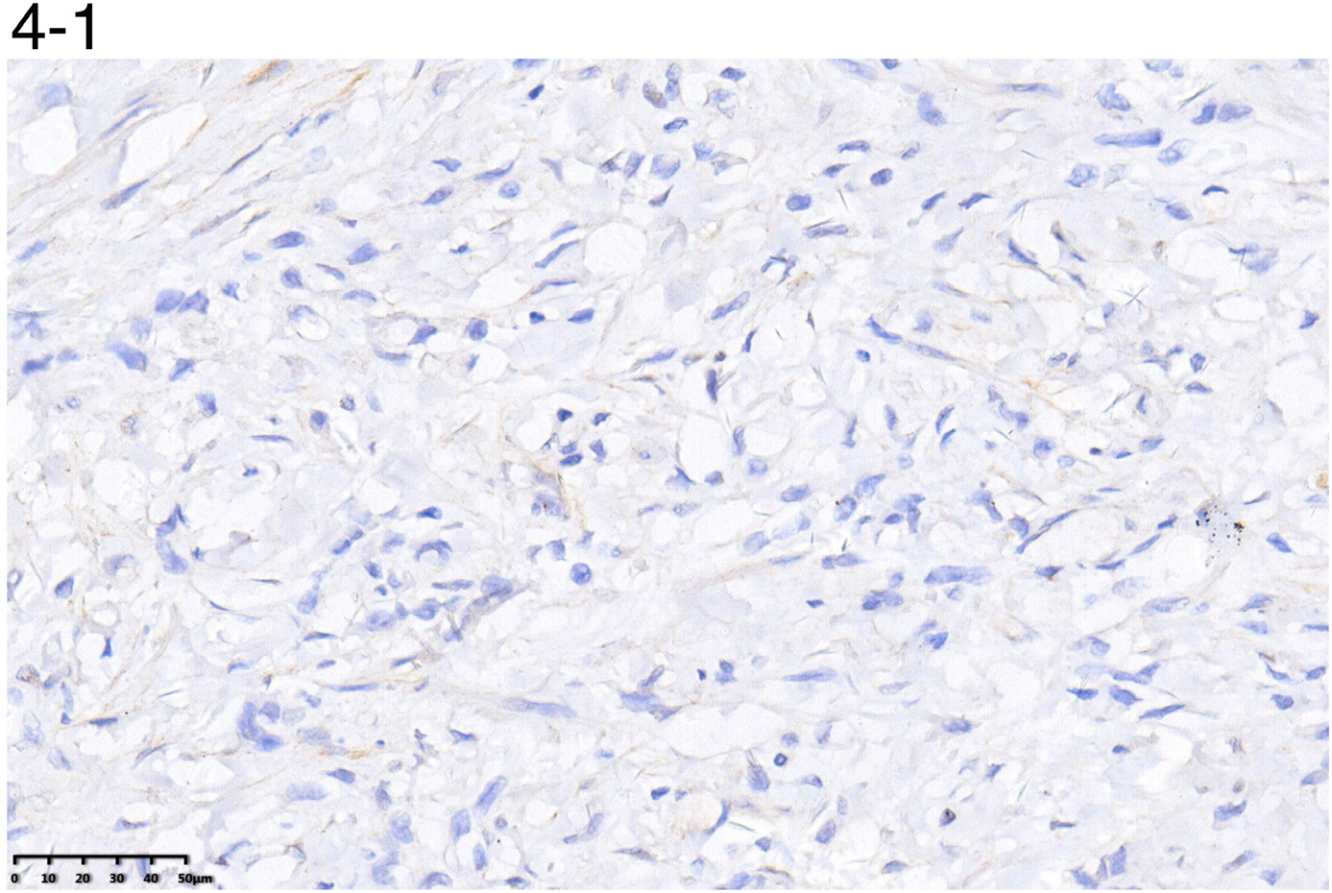

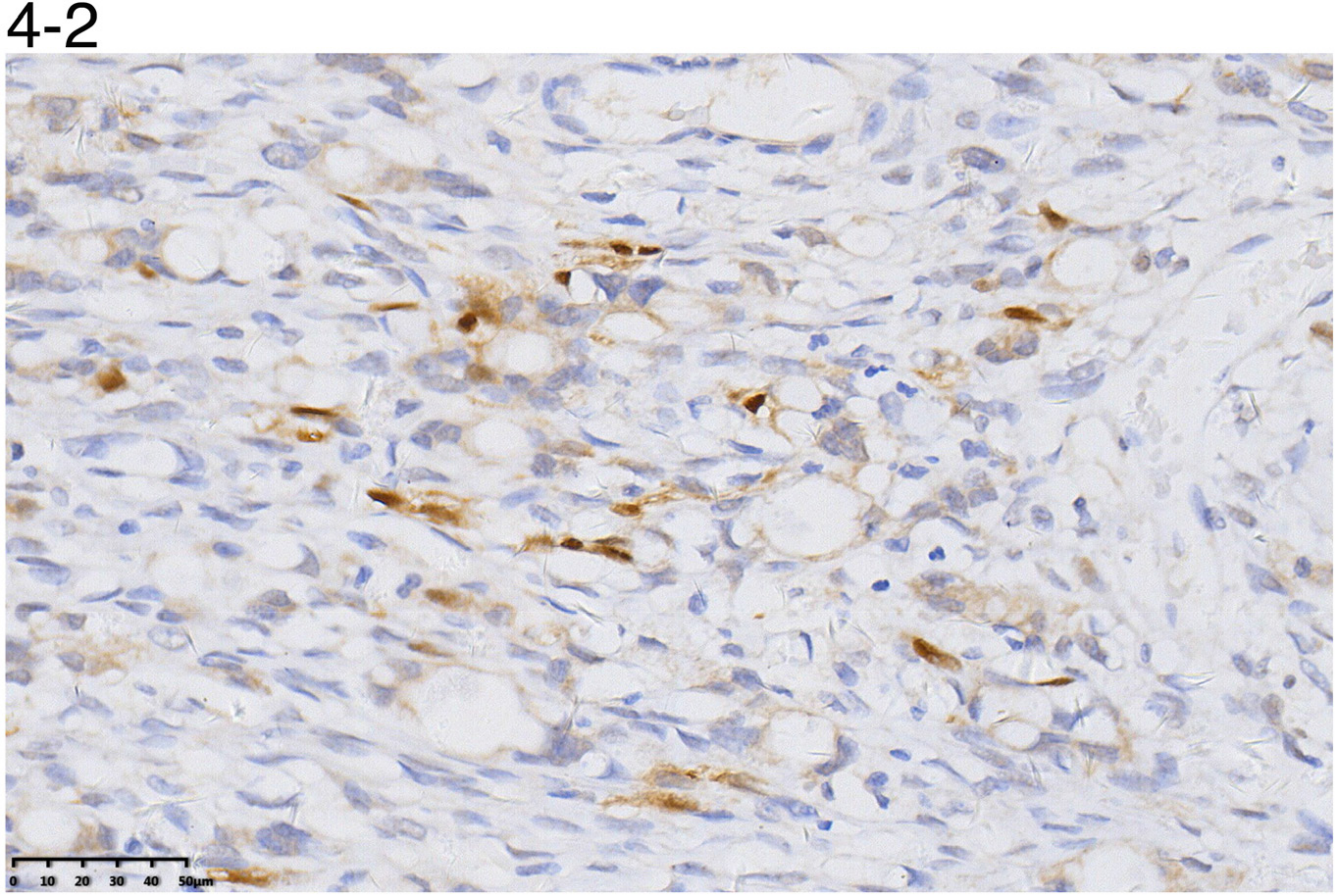
A representative image of negative (4-1) or positive (4-2) ER-β expression

**Figure 5:**
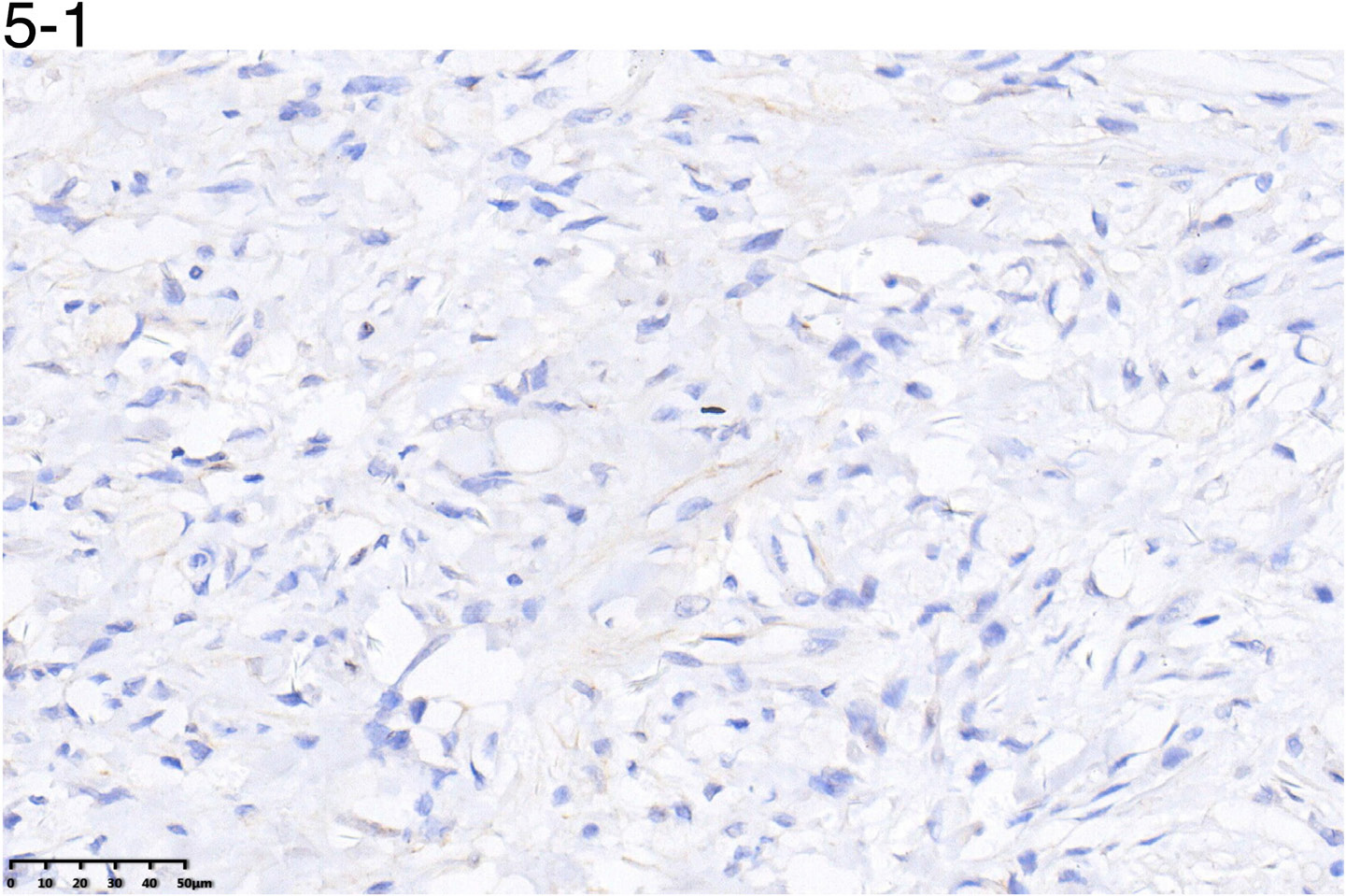

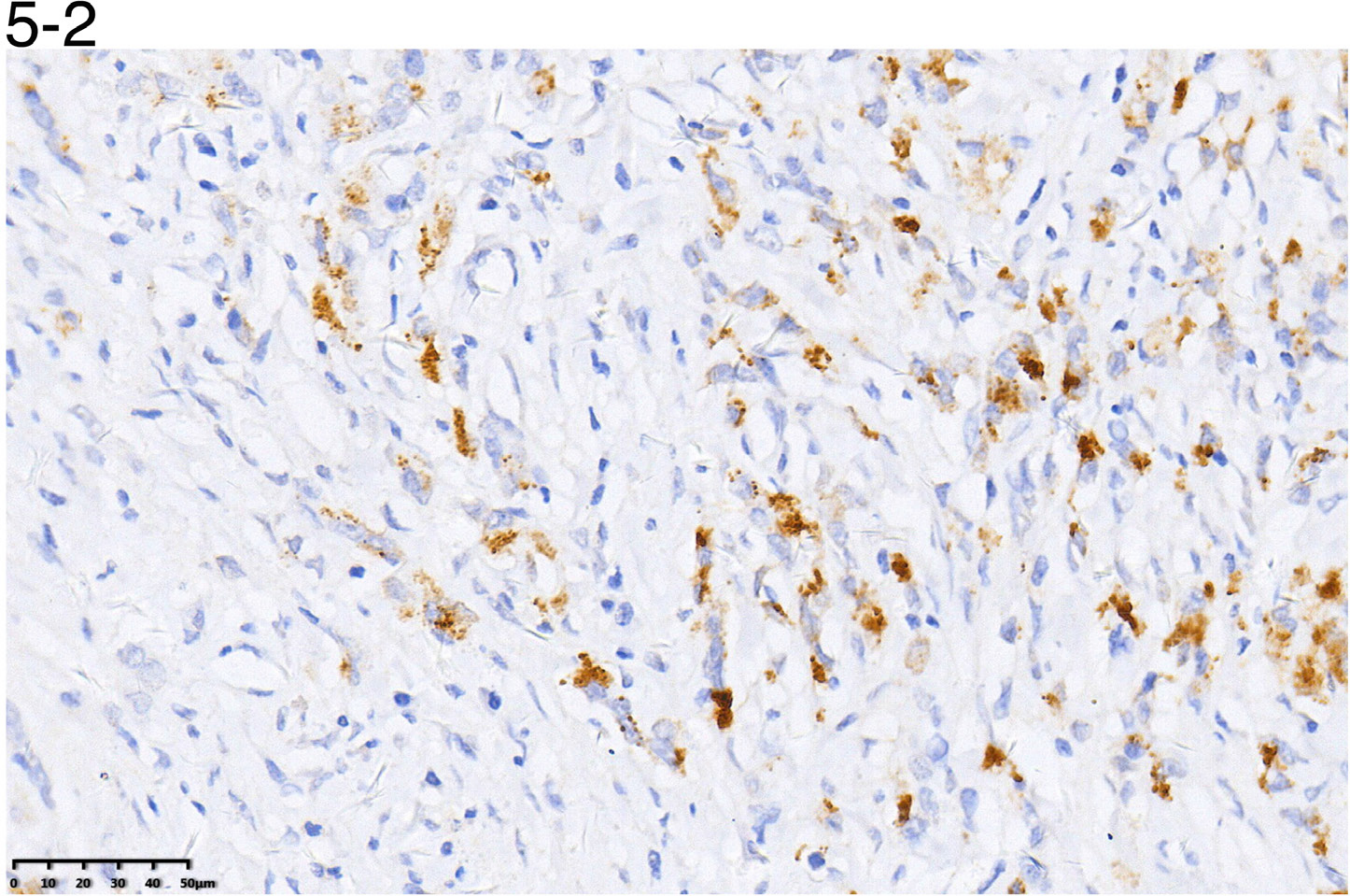
A representative image of negative (5-1) or positive (5-2) PR expression

**Figure 6:**
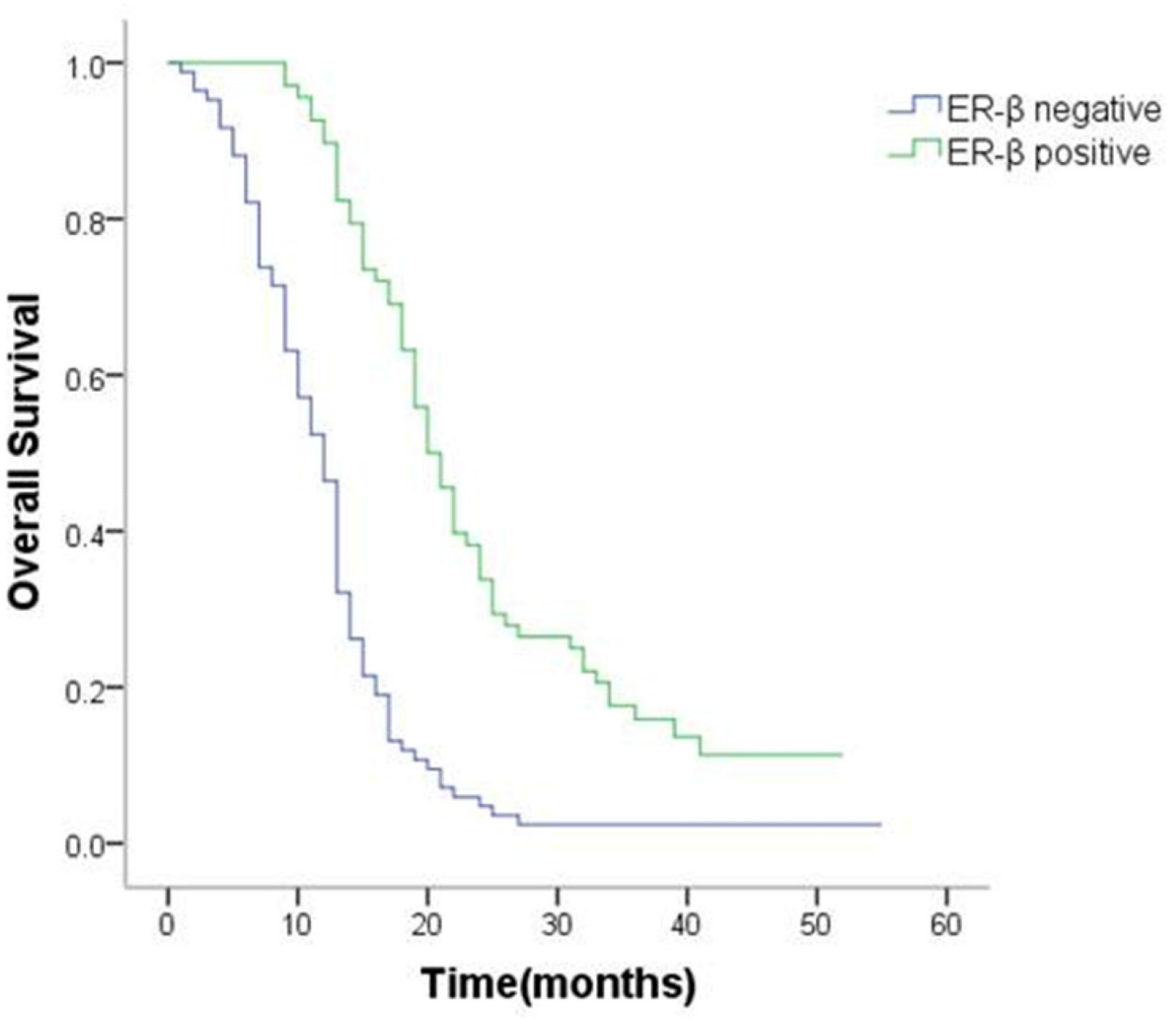
Survival curves of patients with positive or negative expression of ER-β

**Figure 7:**
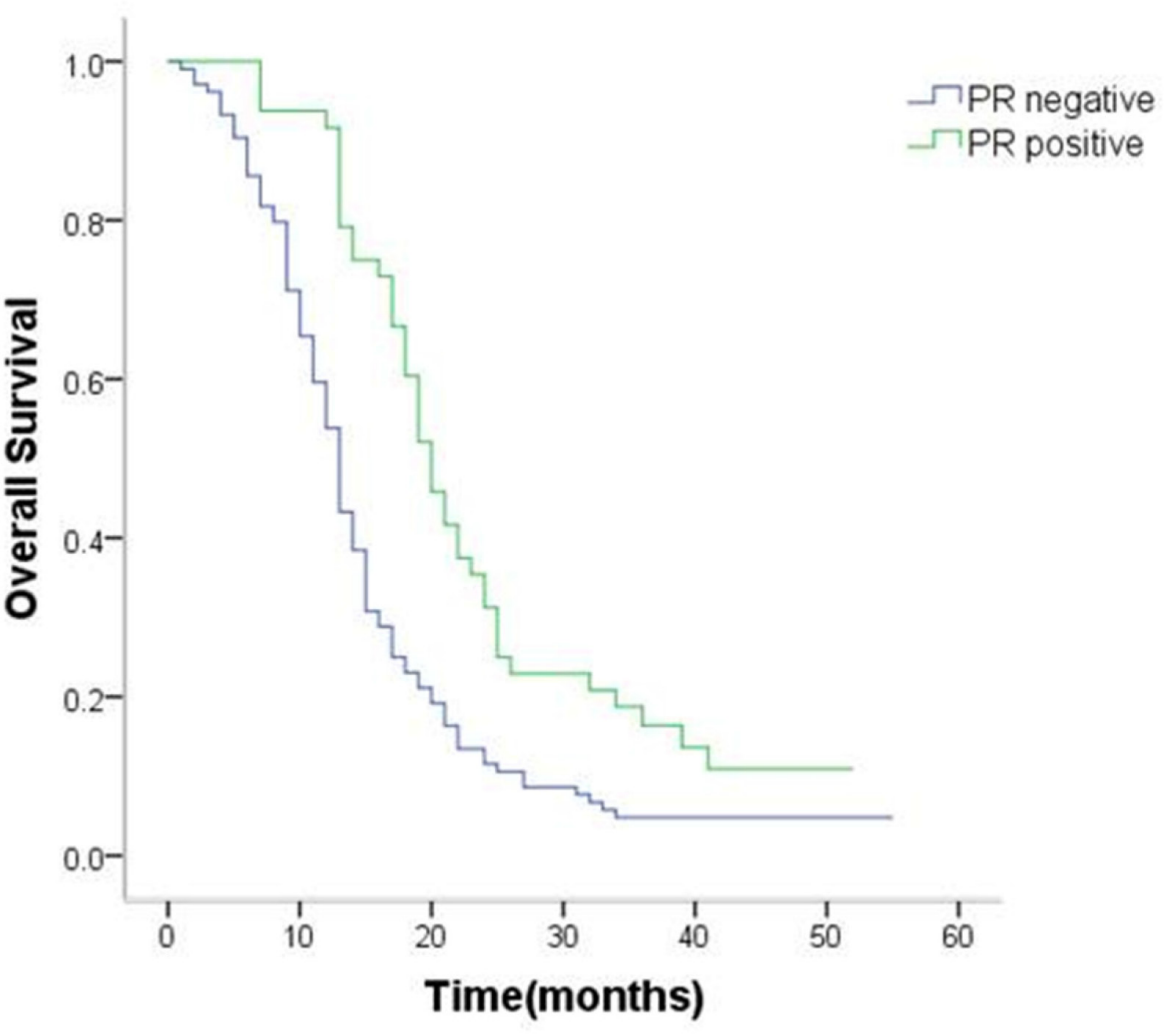
Survival curves of patients with positive or negative expression of PR

The positive/negative expression profiles of representative sex hormone receptors are shown in Figures 4 and 5. Uni- and multivariate analyses showed a positive correlation between expression of ER-β and PR and better survival. The average OS in ER-β-positive and -negative patients was 24.4 (95% CI 21.5–27.4) months and 12.6 (95% CI 10.8–14.4) months, respectively (*P* < 0.001; Figure 6). Average OS in PR-positive and -negative patients was 23.7 (95% CI 20.2–27.3) months and 15.4 (95% CI 13.2–17.5) months, respectively (*P* < 0.001; Figure 7). These results indicate that expression of ER-β or PR are favorably associated with better prognosis in patients with ovarian metastases from a primary gastric cancer.

Furthermore, we evaluated the relationship between the expression of ER-β, PR, and clinicopathologic factors, and found negative expression of ER-β to be significantly associated with peritoneal metastasis, signet-ring cells, and ascites; negative expression of PR was significantly associated with laterality as well as peritoneal metastasis [Table 4]. On multivariate analysis, peritoneal metastasis was significantly associated with negative expression of ER-β and PR. In addition, laterality was also significantly associated with negative expression of PR.

**Table 4:** Correlation between ER-β and PR expressions and clinicopathologic characteristics.

	ER-β			PR		
	(+)	(-)	*P*	(+)	(-)	*P*
Age (years)						
≥50	16	22	0.706	16	22	0.107
<50	52	62		32	82	
Tumor size (cm)						
≥5	48	58	0.837	38	68	0.086
<5	20	26		10	36	
Laterality						
Bilateral	50	57	0.446	41	67	0.008
UnilateraI	18	27		7	37	
Peritoneal metastasis						
No	57	44	<0.001	40	61	0.003
Yes	11	40		8	43	
Signet-ring cells						
Positive	26	46	0.042	21	51	0.544
Negative	42	38		27	53	
Ascites						
No	30	23	0.031	19	34	0.407
Yes	38	61		29	70	
Ovarian metastases						
Synchronous	41	52	0.839	28	65	0.624
Metachronous	27	32		20	39	
Serum CEA (ng/mL)						
Normal	58	61	0.059	38	81	0.859
>5	10	23		10	23	
Serum CA19-9 (U/mL)						
Normal	43	53	0.986	31	65	0.805
>39	25	31		17	39	
Serum CA125 (U/mL)						
Normal	27	25	0.199	14	38	0.373
>35	41	59		34	66	

### Chemotherapy regimens and toxicity

Paclitaxel and S-1 were the most frequently used chemotherapeutic drugs. Median chemotherapy duration was longer in the metachronous group (4.5 cycles, range 3–8 cycles) as compared to the synchronous group (3.7 cycles, range 1–7 cycles) although the difference was not statistically significant (*P* > 0.05). Drug-related adverse events and grade 3 or 4 toxicity occurred in 67.1% (102/152) and 37.5% (57/152) of patients, respectively. The most common grade 3 or 4 hematological toxic effects were leucopenia/neutropenia(26.3%) and thrombocytopenia(7.9%); the most common grade 3 or 4 nonhematological toxic effects were elevated serum aspartate aminotransferase levels (4.6%) and rash (2.6%).

### Postoperative complications

Fifteen patients (16.9%) developed postoperative complications, including anastomotic leakage (*n* = 5), abdominal abscesses (*n* = 4), delayed gastric emptying (*n* = 4), pneumonia (*n* = 2), and bleeding (*n* = 1).Most of these patients were conservatively managed with a successful outcome, but reoperation was necessary in one patient due to intra-abdominal bleeding.

## DISCUSSION

Cancers of the gastrointestinal tract are most likely to generate ovarian metastases in female patients and this is one of the most important causes of treatment failure [11]. The prognosis of patients with gastric cancer and metastasis to the ovaries has been reported to be poorer, as compared with prognosis in other primary gastrointestinal tumors [12]. In the past, chemotherapy was the main treatment for gastric cancer with ovarian metastasis, but the efficiency was disappointing, with a median survival time of 7 to 11 months [13]. Some retrospective study reports in recent years indicated that metastasectomy of Krukenberg tumors could improve prognosis of these patients [7, 8, 9]. However, an optimal therapeutic strategy for Krukenberg tumors from gastric cancer has not been fully established.

Lu et al. retrospectively reviewed a series of 85 patients diagnosed with advanced gastric cancer together with Krukenberg tumor between 2000 and 2010, and reported a median survival time of 14.1 months in the resection group as compared to 8 months in the nonresection group [7]. In another paper, Cho et al. reported that OS differed significantly between patients undergoing metastasectomy plus chemotherapy and those undergoing chemotherapy alone (18.0 months vs. 8.0 months in patients with stage IV gastric cancer; 19.0 months vs. 9.0 months in patients with recurrent Krukenberg tumors).Nevertheless, the imbalance between the two groups should cast some doubts on the significance of the findings [8].

In the present study, we retrospectively analyzed outcomes of 152 patients with synchronous and metachronous ovarian metastasis. Metastasectomy, relative to non-metastasectomy treatment, resulted in significantly better OS in patients with synchronous (19.0 months vs. 13.5 months; *P* < 0.001) and metachronous (24.6 months vs. 14.3 months; *P* = 0.02) ovarian metastasis. The present series included a large study sample, with similar clinicopathologic characteristics for both study groups. Our results are in accordance with those reported in the earlier literature, therefore, we can conclude that metastasectomy should be recommended in patients with ovarian metastasis.

Analysis of prognostic factors can, moreover, facilitate identification of patients most likely to benefit from treatment. Metastasectomy, peritoneal carcinomatosis, and expression of ER-β and PR were independent predictors of OS. Krukenberg tumors are often associated with different degrees of peritoneal metastasis, which frequently induces ascites, intestinal obstruction, or hydronephrosis and seriously impair patient quality of life [14]. There is currently no standard treatment for ovarian metastasis when it presents with peritoneal metastases from gastric cancer, however, cytoreductive surgery in combination with chemotherapy could offer a survival advantage for these patients [15, 16]. In our study, limited peritoneal metastases were resected in a metastasectomy, and this was followed by systemic chemotherapy. Subgroup analysis showed that the survival of the R0 resection group was superior to that of the non-R0 resection group. Therefore, surgical resection without gross residual disease may improve prognosis in patients with Krukenberg tumors. Chemotherapy is another main treatment for these patients, and improves quality of life and OS [17]. Several chemotherapeutic agents are effective against Krukenberg tumors with peritoneal metastasis, including fluoropyrimidine, platinum, taxanes, and epirubicin, either alone or in combination [18, 19]. A European study reported encouraging results from the use of hyperthermic intraperitoneal chemotherapy (HIPEC) [5], but these need to be validated in future research.

Some studies have reported a correlation between expression of sex hormone receptors and the incidence and progression of gastric cancer [20]. It has been suggested that sex hormones play critical protective roles in female patients with gastric cancer [21, 22]. Recently, a meta-analysis suggested that longer exposure to the effects of estrogen may potentially decrease the risk of gastric cancer [23]. However, the presence and role of sex hormone receptors in Krukenberg tumors were undetermined. We evaluated the expression of ER-a, ER-β, and PR in gastric cancer specimens, and found that only ER-β and PR were detected. Our data showed that positive expression of ER-β or PR were favorably associated with better prognosis of gastric cancer patients with ovarian metastases. From our results and those reported in the related literature, ER and PR play an important role in the etiopathogenesis of Krukenberg tumors. However, some issues remain to be resolved. It is unclear whether targeted therapy of sex hormone receptors can lower the incidence of ovarian metastasis from ER- or PR-positive gastric cancer. Moreover, the possibility of ER or PR expression being used as a reliable indicator of prophylactic ovariectomy is worth exploring. Thus, future studies investigating the significance of ER or PR expression in carcinogenesis and tumor progression of gastric cancer and Krukenberg tumor are necessary.

Our study had certain limitations because it was a retrospective analysis, and several confounding factors may have influenced our findings. However, the fact that it reports results from a large series of patients and presents the first evaluation of the role of sex hormone receptors in Krukenberg tumors from gastric cancer. The results are expected to provide useful information for formulating treatment strategies for those patients.

The present study demonstrated that the metastasectomy in combination with chemotherapy was associated with survival benefits in patients with synchronous or metachronous Krukenberg tumors from gastric cancer. Metastasectomy, peritoneal carcinomatosis, and ER-β and PR expression status were prognostic factors for survival. Well-designed prospective studies are needed to confirm these results, and will be important in developing optimal treatment strategies for Krukenberg tumors of gastric origin.

## MATERIALS AND METHODS

### Patients

From January 2005 to December 2014, 4381 female gastric cancer patients were admitted and treated at the Zhejiang Cancer Hospital. Among these patients, 152 with krukenberg tumor detected by the imaging studies or by pathological evaluation of the metastasectomy specimens were retrospectively reviewed. Patient demographics, radiological details, surgical data, pathological features, and survival were collected and analyzed. Outpatient records combined with telephone interviews were used for follow-up. All the patients suspected of having Krukenberg tumor underwent imaging studies to identify the extent of disease and resectability. The patients included in this study were initially regarded as having resectable diseases. Therefore, a resection was only performed in case of Krukenberg tumor without or with limited peritoneal dissemination. At the completion of surgery, the residual disease state of each patient was recorded according to the presence or absence of gross residual disease, which was classified as negative resection margins (R0), microscopic tumor infiltration (R1), and macroscopic residual tumor (R2).

Overall, 93 patients were initially diagnosed as synchronous metastasis and 59 as metachronous metastasis after they underwent curative resection of gastric cancer. Patients were divided into two groups according to treatment modality: the metastasectomy group( received both chemotherapy and metastasectomy for Krukenberg tumor) and the non-metastasectomy group(received chemotherapy alone). Overall survival (OS) of patients with synchronous ovarian metastasiswas defined as duration from the date of pathologic diagnosis of gastric cancer to the date of death or last follow-up. OS of patients with metachronous ovarian metastasis was defined as duration from the date of radiologic diagnosis of Krukenberg tumor to the date of death or last follow-up. For both groups, December 31, 2016 was the cutoff date for OS.

### Detection of sex hormone receptors in gastric cancer specimens

The expression of estrogen receptor (ER)-α,ER-β, and progesterone receptor (PR) in gastric cancer, which may potentially be related to the ovarian metastasis, was detected by immunohistochemistry (IHC) using the Novolink Polymer Detection System as described previously [10]. The following primary antibodies were used: ER-α (ab37438, dilution 1:200; Abcam, Cambridge, UK), ER-β (ab288, dilution 1:100; Abcam), and PR (ab16661, dilution 1:100; Abcam). All specimens were independently evaluated by two pathologists who were blinded to study grouping. Samples where more than 10% of the tumor cells were stained were regarded as positive.

### Ethics statement

The study was approved by the institutional ethics review board of Zhejiang Cancer Hospital. Written informed consent was obtained from all study participants. All study procedures were undertaken in accordance with the principles of the Declaration of Helsinki and relevant human research policies in China.

### Statistical analysis

For continuous variables, two-tailed Student *t*-tests were used to compare demographic and clinical characteristics between the study groups. For discrete variables, a chi-square test was used. Kaplan–Meier analysis was used to calculate survival, and log-rank test was performed to compare OS between treatment groups. Independent prognostic factors were determined by multivariate analysis using the Cox proportional hazards model. All statistical analyses were conducted on SPSS 19.0 for Windows (IBM Corporation, Armonk, NY, USA). A *P* value less than 0.05 was considered indicative of statistical significance.

## Abbreviations

OS: overall survival
HIPEC: hyperthermic intraperitoneal chemotherapy
ER: estrogen receptor
PR: progesterone receptor
IHC: immunohistochemistry
